# Cellular reprogramming to model and study epigenetic alterations in cancer

**DOI:** 10.1016/j.scr.2020.102062

**Published:** 2020-12

**Authors:** Jungsun Kim

**Affiliations:** Department of Molecular and Medical Genetics, Cancer Early Detection Advanced Research Center, Knight Cancer Institute (Cancer Biology Research Program), Oregon Health & Science University School of Medicine, KCRB 5001.51, 2720 SW Moody Ave., Portland, OR 97201, United States

## Abstract

Although genetic mutations are required for cancer development, reversible non-genetic alterations also play a pivotal role in cancer progression. Failure of well-orchestrated gene regulation by chromatin states and master transcription factors can be one such non-genetic etiology for cancer development. Master transcription factor-mediated cellular reprogramming of human cancer cells allows us to model cancer progression. Here I cover the history and recent advances in reprogramming cancer cells, followed by lessons from cellular reprogramming of normal cells that may apply to cancer. Lastly, I share my perspective on cellular reprogramming for studying epigenetic alterations that have occurred in tumorigenesis, discuss the current limitations, and propose ways to overcome the obstacles in the reprogramming of cancer.

## Highlights

• Cellular reprogramming to model human cancer.
• Cellular reprogramming to rewire epigenetic alterations in human cancer.
• Selective reactivation of malignancy in the cell lineage cancer is originated.
• Cellular reprogramming to recapitulate human cancer progression.

## 1 Introduction

Genetic alterations of oncogenes and tumor suppressors are critical drivers of tumorigenesis. However, evidence now shows that epigenetic changes—both intrinsic and extrinsic to cells—may drive malignancy, supporting the possibility of cancer reversibility. The first evidence for cancer reversibility was shown in the pioneering work of Dr. Braun with crown-gall tumors of plants (Braun, 1951, Braun, 1959) in the 1950s, around the time the DNA double helix was discovered and Waddington's “epigenetic landscape” was introduced (Waddington, 1957). By performing serial grafts of teratoma tissues of single-cell origin to the stem ends of healthy tobacco plants with the axillary bud removed, he demonstrated gradual recovery of teratoma cells to normal, flowering, and ultimately setting seed. He proposed that, rather than somatic mutations, the “uncharacterized cytoplasmic entity” responsible for the cellular alteration of crown gall tumor cells could be an autonomous or partially autonomous factor that was influenced by dilution in rapidly dividing cells (Braun, 1959). Subsequently, advances in molecular developmental biology techniques in the 1960-1980s enabled researchers to pinpoint the reversible non-genetic factors and establish the concept more firmly. The more recent breakthrough discovery of induced pluripotent stem cells (iPSCs) (Takahashi and Yamanaka, 2006, Takahashi et al., 2007) advanced the knowledge one step further in human cancer and translated into a variety of potential applications in cancer biology, including understanding cancer progression and early disease, and developing new biomarkers.

In this review, I give a concise overview of the past, present, and future of cellular reprogramming to understand and model human cancer. I first summarize the historical evidence for cancer reversibility in mammalian cells by blastocyst injection, cell fusion, and nuclear transplantation experiments. I then briefly describe the basic concept of cellular reprogramming in normal somatic cells and discuss the up-to-date advances on cellular reprogramming of various cancers. I compare similar and distinctive aspects of cancer development and cellular reprogramming, and lastly discuss the prospects of cellular reprogramming for neoplastic disease along with the challenges associated with iPSC-based approaches in cancer.

## 2 History of experimental evidence of cancer reversibility in animals

The altered interplay between genetic and epigenetic networks contributes to tumorigenesis (Baylin and Jones, 2016). Yet, in rare examples, epigenetic alterations have been shown sufficient to initiate tumorigenesis prior to or without driver mutations (Baylin and Jones, 2016, Esteller et al., 2001, Holm et al., 2005, Sakatani et al., 2005). Is rewiring such epigenetic alterations enough to control the cancerous phenotype?

Early attempts to control the cancerous phenotype in mammals were made in murine teratocarcinoma cells by blastocyst injection in the 1970s (Brinster, 1974, Mintz and Illmensee, 1975, Illmensee and Mintz, 1976). Dr. Brinster transferred teratocarcinoma cells (taken from ascites fluid of agouti mice) into blastocysts from Swiss albino mice (Brinster, 1974). These blastocysts developed into 60 adult mice, all of which maintained the skin graft derived from the agouti mice for significantly longer than uninjected control animals, indicating the true formation of chimeric mice. One of the males in this group had small patches of agouti hair on his body yet failed to produce offspring. Thus, it was suggested that the “embryo environment can bring the autonomous proliferation of the teratocarcinoma cells under control” (Brinster, 1974).

To test the developmental consequences of genetic variations occurring in malignant carcinoma cells, Dr. Mintz injected single euploid teratocarcinoma cells (derived from the core of embryoid bodies grown as an ascites tumor) into blastocysts bearing many genetic markers (Illmensee and Mintz, 1976); 44% of the blastocysts survived, and all were mosaic with 129-strain cells, which was the background strain of the teratocarcinoma cells. The distribution of 129-strain cells was sporadic in developmentally unrelated tissues and many genes that had been undetectable in the original tumors were expressed, indicating the restoration of orderly gene expression and cessation of the proliferation of euploid malignant tumors in a normal embryonic environment (Mintz and Illmensee, 1975, Illmensee and Mintz, 1976). All these transplanted teratocarcinoma cells were euploid; thus, it was suggested that the euploid genome was required for the normal development of malignant cells (Illmensee and Mintz, 1976).

While these early chimeric studies indicated that the embryonic environment could maintain control of the aberrant proliferation of teratocarcinoma cells, the studies used euploid teratocarcinoma cells, which are pluripotent, and efficiency was very low. Thus, it was not clear whether somatic cancer of aneuploidy could be reprogrammed. The first experiment of reprogramming of aneuploid tumors was performed in frogs (McKinnell et al., 1969) in the 1960s by nuclear transplantation (NT) (Gurdon et al., 1958). NT triggers a reversal of the epigenetic status established during cellular differentiation. Triploid nuclei of renal tumor cells were transplanted into enucleated eggs. A small fraction of the transferred triploid tumors (32 of 143) developed partial and complete blastulas. A quarter of these blastulas further developed to swimming embryos with a similar efficiency level in their development to diploid transplants (McKinnell et al., 1969). Analysis of living tadpoles and embryos confirmed various functional tissues of the triploid genome (McKinnell et al., 1969). However, it was later found that tadpoles lacked the Lucke tumor herpesvirus DNA fragment, which is the neoplastic etiological agent (Carlson et al., 1994). Together, the data suggested that aneuploidy didn't interfere with nuclear transplantation, development of the cloned embryo, or the formation of functional tadpoles. However, since the tadpoles’ genomes excluded the genetic neoplastic etiological agent by unknown mechanisms, whether genetic etiology could predispose the cloned animals to subsequent malignancy remained untested.

In the early 2000s, several studies showed examples of both reprogramming of the cancer genome into pluripotency and predisposition of cloned embryos or animals to malignancy using NT in a subset of murine tumors (Blelloch et al., 2004, Hochedlinger et al., 2004, Li et al., 2003).

Medulloblastoma is the most common pediatric brain tumor and originates from primitive neuroepithelial cells in the developing cerebellum (Li et al., 2003). To determine to what extent epigenetic reprogramming can reverse oncogenesis, Li et al. transferred the nuclei of medulloblastoma arising in *Ptc1^+/-^* mice into enucleated eggs (Li et al., 2003). Cloning efficiency was lower than for control nuclei of normal spleens; however, 10% of cloned eggs with nuclei of medulloblastoma developed into morulae or blastocyst stages. These were morphologically indistinguishable from normal spleen-derived nuclei and showed no evidence of uncontrolled cell growth characteristic of cultured tumors (Li et al., 2003). To test whether cloned blastocysts can direct later stages of development, these were transplanted into pseudopregnant females. The resulting embryos appeared grossly normal and developed all three germ layers of cells until embryonic day 7.5 (E7.5). Intriguingly, embryos at E8.5 showed extensive differentiation toward cephalic vesicles and neural tube, and no viable embryos were observed after E8.5 (Li et al., 2003). This demonstrated that the oncogenic pathways that cause medulloblastoma were reprogrammed and silenced to some extent until gastrulation. They were then reactivated within the context of the cerebellar granule cell lineage, which ultimately resulted in embryonic lethality.

Similar reactivation of tumorigenicity during embryogenesis was also observed in a study using embryonal carcinoma (EC) cells (Blelloch et al., 2004), an embryonic tumor originating from primordial germ cells (Stevens, 1970, Martin, 1980), which originate from the early post-implantation epiblast cells during primitive streak formation (Chiquoine, 1954). Blelloch et al. transferred nuclei from three independent EC cells into enucleated eggs (Blelloch et al., 2004). All clones were able to direct early embryo development and produced morphologically normal blastocysts that gave rise to ES cells at high efficiency. However, the resulting EC-nuclear transplanted ES cells retained the same potential as their parental donor EC cells in late developmental and tumorigenic potential (Blelloch et al., 2004). Thus, this study suggested that reactivation of the tumorigenic potential of EC-NT ES cells within the context of the parental EC cells occurred after gastrulation.

In contrast to embryonic origin tumors, adult melanoma tumor nuclei-transferred embryos were developed into chimeric mice (Hochedlinger et al., 2004). Hochedlinger et al. transplanted nuclei from various tumor cell types into enucleated oocytes (Hochedlinger et al., 2004). In line with other studies, most eggs with tumor nuclei underwent normal pre-implantation development until the blastocyst stage, although those with nuclei from *p53^-/-^* breast cancer and *Ras^+^/Ink4a/Arf^-/-^* fibroblasts developed more efficiently. Blastocysts derived from one RAS-inducible melanoma cell line (*Ras^+^/Ink4a/Arf^-/-^*) were further able to generate ES cells, which were capable of differentiation into multiple somatic cell types *in vivo* (Hochedlinger et al., 2004). This indicated that melanoma NT-ES cells reset the cancer epigenetic state and acquired pluripotency to differentiate into tissues of various lineages. These melanoma NT-ES cells retained a genetic alteration (trisomy 8 with 8qter deletion) from their parental melanoma, confirming the origin of the NT-ES line. However, when melanoma-NT-ES cells were injected into a tetraploid blastocyst, the embryos were able to develop multiple functional tissues only up to E9.5, suggesting that the melanoma genome could not provide an organizational framework for late cell specification. Following injection into blastocysts, the melanoma-NT-ES cells generated chimeric mice, which eventually developed multiple primary melanoma lesions with higher penetrance and an expanded tumor spectrum compared to the murine donor model. Moreover, 33% of the chimeras developed rhabdomyosarcoma, the incidence of which is frequently correlated with a loss of the chromosome 8q region, suggesting a potential overlap between the pathways that operate in melanoma and rhabdomyosarcoma (Hochedlinger et al., 2004). Thus, the authors concluded that the melanoma genomes are compatible with a broad developmental potential yet predispose mice to malignant tumors.

Together, these early studies led to the following conclusions: a) Oocyte cytoplasm can, to some extent, reset accumulated epigenetic modifications associated with genetic mutations and cell lineages, and keep malignant phenotypes under control until the blastocyst stage, regardless of cancer type (despite variable cloning efficiency caused by unknown factors); b) During late cell specification, irreversible genetic alterations inherited by the donor nucleus contribute to reactivation of tumorigenic potential within the context of specific cell lineages where mutations can be expressed to oncogenic pathways. These observations suggest that nuclear reprogramming strategies can be harnessed to model cancer progression. However, so far, no human cancers have been successfully reprogrammed by NT.

Several studies in the 1970s and 80s also showed malignancy suppression by fusion with healthy somatic cells without reprogramming cancer cells into pluripotency. Yet, normal cells' ability to reverse tumorigenicity in malignant cells depended on gene dosage and cell types (Harris, 1988). For example, the human aneuploid cell line Hela could not activate muscle genes when fused with muscle cells. Pretreatment of cells with an inhibitor of DNA methylation rescued this refractory status (Chiu and Blau, 1985). This indicated that, in contrast to massive reprogramming processes like NT, cell fusion was not enough to remove aberrant epigenetic changes accumulated in human aneuploid cells or to reactivate cell-type-specific genes.

## 3 Reprogramming of cancer by adapting induced pluripotent stem cell methodology

A defined set of master transcription factors (TFs), namely OCT4, SOX2, KLF4, and MYC (called “OSKM” hereafter), can reprogram somatic cells into induced pluripotent stem cells (iPSCs) and allow the study of dynamic events that occur during cell fate decisions (Takahashi and Yamanaka, 2006, Takahashi et al., 2007). Since TF-mediated cellular reprogramming does not require blastocysts or eggs, it avoids ethical issues and has indeed been widely adopted in many labs to model human disease. As a result, the reprogramming of various human cancers has been re-visited utilizing this cellular reprogramming methodology.

Cancer modeling using iPSC methodology is most advanced for blood cancers, including human (Chao et al., 2017, Lee et al., 2017, Kotini et al., 2017) or murine (Liu et al., 2014) acute myeloid leukemia; myelodysplastic/myeloproliferative neoplasms (Kotini et al., 2017, Kotini et al., 2015, Ye et al., 2009, Ye et al., 2014, Saliba et al., 2013, Gomez Limia et al., 2017, Takei et al., 2018, Hsu et al., 2019); chronic myeloid leukemia (CML) starting with primary cells (Hu et al., 2011, Kumano et al., 2012, Amabile et al., 2015, Miyauchi et al., 2018, Suknuntha et al., 2015) or a CML cell line (Carette et al., 2010); juvenile myelomonocytic leukemia (Gandre-Babbe et al., 2013, Mulero-Navarro et al., 2015, Gagne et al., 2018, Tasian et al., 2019), acute lymphocytic leukemia (Munoz-Lopez et al., 2016); and genetic predisposition disorders (Sakurai et al., 2014, Suzuki et al., 2015). A handful of solid tumors have been reprogrammed to model cancer, including pancreatic cancer (Kim et al., 2013, Khoshchehreh et al., 2019), gastrointestinal (GI) cancer cell lines (Miyoshi et al., 2010, Ogawa et al., 2015); glioblastoma (Stricker et al., 2013), sarcoma (Zhang et al., 2013), Li-Fraumeni syndrome (Lee et al., 2015), melanoma (Bernhardt et al., 2017), lung cancer cell lines (Zhao et al., 2015), and plexiform neurofibromas (Carrio et al., 2019). The cellular reprogramming studies of blood cancers inherited familial predisposition and Li-Fraumeni Syndrome are discussed elsewhere in this issue. Here, I highlight some of the studies of cellular reprogramming of solid tumors and their potential application in cancer biology.

### 3.1 Reprogramming of human melanoma cell lines and gastrointestinal (GI) cancer cell lines

Bernhardt et al. reprogrammed human melanoma cell lines harboring *BRAF^V600E^* or *NRAS* mutations with TetO-inducible OSK (Bernhardt et al., 2017). These reprogrammed iPSC-like cells (“induced pluripotent cancer cells, iPCCs”) were dependent on the expression of exogenous OSK factors, showing a metastable pluripotent state. Nevertheless, iPCCs developed teratomas that did not contain melanoma cells. *In vitro*, iPCCs were capable of terminal differentiation into different cell lineages, such as neurons and fibroblast-like cells. Notably, while all parental cell lines were sensitive to MAPK inhibitors, the iPCCs and their differentiated fibroblast cells showed resistance to MAPK inhibitors. Cancer growth and survival can be impaired by the inactivation of a few oncogenic pathways. This phenomenon, called oncogene addiction, is utilized for targeted molecular therapies (Weinstein and Joe, 2006). It would be interesting to examine whether oncogenic addiction persists in iPCC-derived melanoma cells, although most teratoma tissues lacked melanoma cells. Similar observations were made in other CML iPSC studies (Kumano et al., 2012, Carette et al., 2010). Human CML-derived iPSCs or their non-hematopoietic lineage differentiated cells also showed a loss of BCR-ABL dependence (Kumano et al., 2012, Carette et al., 2010). Hence, a better understanding of the molecular basis of the events that counteract oncogenic pathways in iPSCs and their differentiated cells could provide insight into resistance to targeted therapies and loss of oncogene addiction as a result of altered cellular states.

In contrast, another study showed increased sensitivity of cancer iPSCs and their differentiated cells to antimetabolite therapy and differentiation-inducing treatment. Miyoshi et al. reprogramed the colorectal cancer cell line DLD-1 to pluripotency using OSKM retroviral cocktails (Miyoshi et al., 2010). The induced pluripotent cancer cells (called “iPC”) expressed a subset of pluripotency markers and could be differentiated into embryoid bodies. This was followed by adherent differentiation, at which point they were called “PostiPC” and expressed markers of the three germ layers. Proliferation was reduced in iPC but not in PostiPC, compared to the parental DLD-1 cell line. Likewise, there were no differences in invasive capacity between PostiPC and the parental cell line (Miyoshi et al., 2010). Invasiveness was not measured in iPC cells, so it is unclear whether this invasive ability was regained in PostiPC or persisted through iPC stages.

Nevertheless, in the presence of differentiation agents, such as retinoic acid and vitamin D3, both invasiveness and proliferation of PostiPC were significantly reduced, compared to the parental DLD-1 cell line. PostiPC cells acquired sensitivity to the anti-cancer drug 5-fluorodeoxyuridine to a higher degree than parental DLD-1 cells, showing that reprogramming of cancers could restore sensitivity to anti-cancer agents. Consistently, PostiPC showed reduced tumor formation compared to parental DLD-1 cells in an *in vivo* xenograft assay, indicating reduction of tumorigenicity via reprogramming (Miyoshi et al., 2010). Thus, this study showed the potential application of cancer cell reprogramming as a therapeutic to sensitize cancer cells to differentiation and anti-cancer agents.

Together, the above studies demonstrated the potential application of cancer cell reprogramming to the study of resistance or response to cancer therapies influenced by cellular states.

### 3.2 Reprogramming of sarcoma cell lines, glioblastoma (GBM) neural stem cells, and plexiform neurofibromas (PNFs)

Zhang et al. attempted reprogramming various sarcoma cell lines using OSKM, LIN28, and NANOG and achieved a slightly higher reprogramming efficiency (Zhang et al., 2013). These reprogrammed cells expressed standard pluripotency markers and could be directed to differentiate into endoderm and ectoderm yet failed to differentiate spontaneously. Their epigenetic signature revealed that reprogrammed sarcoma cell lines resemble much more mesenchymal stem cells (MSC) than ES cells or sarcomas. Indeed, these cells did not form benign teratoma tissues but exhibited reduced malignant features in a xenograft assay compared to their parental sarcoma cell lines. Reprogrammed sarcoma cell lines were capable of differentiating into other mesodermal lineages, which were blocked in their parental cell lines, and showed abrogated tumorigenicity compared to the parental sarcoma cells.

Although the reprogramming process is accompanied by global DNA demethylation, most genes (182/205 promoters) did not show a definitive increase in gene expression (Zhang et al., 2013). This suggests the presence of additional gene expression control mechanisms. Indeed, while parental sarcoma cell lines contained the active histone modification H3K4me3 in their *MYC* promoters, reprogrammed sarcoma cell lines had both active H3K4me3 and repressive H3K27me3 marks in their *MYC* promoter. These marks represent a poised state of developmental genes in ES cells (Taberlay et al., 2011). Reprogrammed sarcoma cells that were differentiated to adipogenic and osteogenic lineages no longer contained the active mark H3K4me3 in the *MYC* promoter. Consistent with this, although all parental sarcoma cell lines expressed a basal level of endogenous *MYC*, *MYC* expression was dramatically decreased in reprogrammed sarcoma cells despite the delivery of ectopic *MYC* during reprogramming.

In summary, Zhang et al. showed that partially reprogrammed sarcoma cancer cell lines (pre-iPS) exhibit reduced tumorigenicity, resemble mesenchymal stem cells (MSC), and can differentiate into lineages that were blocked in their parental cell lines (Zhang et al., 2013). They also showed massive rewiring of epigenetic marks associated with oncogenes and tumor suppressors in the reprogrammed cells.

Stricker et al. also investigated global epigenetic resetting and tumorigenicity in reprogrammed cancer cells (Stricker et al., 2013). Glioblastoma (GBM) is the most prevalent human brain cancer and is driven by an immature GBM stem cell that displays many characteristics of normal neural stem cells (NS). DNA hypermethylation is frequently found at non-classical oncogenes, such as polycomb repressor complex 2 (PRC2), *TES*, and *CDKN1C* in GBM. To study the functional consequences of resetting GBM-associated DNA methylation defects, Stricker et al. reprogrammed glioblastoma (GBM)-derived neural stem cells (GNS) into iPSCs (“GiPSC”), using piggyback transposon-mediated OCT4 and KLF4 (Stricker et al., 2013). All clones developed teratomas, yet these teratomas were immature with highly proliferating cells. Overall, 65% of cancer-specific methylation variable positions (cMVPs) were reset in GiPSC lines.

An in-depth analysis of one GNS cell line along with two independent GiPSC derivatives showed that 85% of PRC2 target genes were associated with cMVPs, and most of these cMVPs (92%) were hypermethylated in cancer (Stricker et al., 2013). Half (53%) of PRC2 target genes were associated with cMVPs that were significantly demethylated during reprogramming. To assess the consequence of resetting cMVPs on tumorigenicity, they subsequently re-differentiated GiPSC to neural progenitors (“GiPSC-N”) as well as non-neural lineages (mesodermal/cartilage, “GiPSC-M”). The majority of cMVPs that were reset during reprogramming persisted in both differentiated GiPSC derivatives (GiPSC-N and GiPSC-M) *in vitro*. These included promoter regions commonly associated with DNA hypermethylation in GBM, such as those of *TES*, *CDKN1C*, and many PRC2 target genes. Only a minority of the cMVPs reacquired methylation in GiPSC-N. Despite widespread resetting of cMVPs in the GiPSCs and their derivate GiPSC-N, GiPSC-N developed infiltrating tumors in *in vivo* xenotransplantation. This indicates that the resetting of cMVPs alone was not sufficient to alter the malignant behavior of GBM tumor-initiating cells. In contrast, GiPSC-M developed benign cartilaginous tumors *in vivo*.

In summary, Stricker et al. demonstrated that reprogramming could reconfigure the widespread resetting of cancer-specific aberrant DNA methylation and that this resetting stably persisted in neuronal and non-neuronal lineage cells. Despite persistent resetting of cMVPs in neuronal and non-neural lineage cells, tumor suppressors were de-repressed, and malignancy was suppressed only in non-neural lineages (Stricker et al., 2013).

Another study also provided evidence of selective activation of tumorigenic properties in the lineage of the parental tumor (Carrio et al., 2019). Benign and early-stage tumors typically carry a small number of mutations, and thus, reprogramming barriers imposed by these genetic alterations may be lesser than those in advanced tumors. PNFs are benign Schwann cell (SC) tumors of the peripheral nerve sheath and mainly develop due to *NF1* inactivation. Carrio et al. generated iPSC lines from PNF primary cells with *NF1* (-/-) alleles (Carrio et al., 2019). *NF1* (-/-) iPSC clones exhibited, on average, a 10%-15% increase in cell proliferation. The *NF1* (-/-) iPSCs were then differentiated into neural crest (NC) lineages from which SCs developmentally originate. NC cells from *NF1* (-/-) iPSCs were not noticeably distinct compared to NC cells from *NF1* (+/+) iPSCs. NC cells give rise to multiple cell lineages, such as peripheral neurons, melanocytes, as well as SC, from which PNFs develop. *NF1* (-/-) iPSC-NC were able to differentiate into peripheral neurons and melanocytes. However, *NF1* (-/-) iPSC-NC showed uncontrolled proliferation capacity even after day 14 of differentiation into SC, whereas control *NF1* (+/+) iPSC-NC arrested their proliferation. *NF1* (-/-) iPSC-NC also exhibited a lack of myelination capacity when co-cultured with dorsal root ganglion neurons in myelination medium (Carrio et al., 2019). Therefore, this study showed that the deletion of *NF1* did not cause abnormal phenotypes during differentiation of NC into other lineages but did alter the phenotypes during cell specification of NC into SC lineages.

Altogether, the above studies showed that TF-mediated cellular reprogramming reset or altered cancer-associated epigenetic changes such as DNA methylation and malignant phenotypes and restored the differentiation potential of reprogrammed cancers. While reset epigenetic changes persisted in the differentiated cells, the suppressed malignant phenotypes were selectively re-activated in the lineages corresponding to the primary cancers, but not in other lineages.

### 3.3 Reprogramming of primary pancreatic ductal adenocarcinoma (PDAC)

Human PDAC has a dismal prognosis, mainly because tumors are usually detected too late to be effectively treated, and reliable early detection markers do not exist. Current human PDAC models capture only the endpoint state of PDAC and do not reflect the events that occur in the early stages of the disease, such as in the putative cancer precursor, pancreatic intraepithelial neoplasia (PanIN).

To address this gap, we employed a reprogramming technology to create an iPS-like cell line from an advanced, recurrent human PDAC (Kim et al., 2013). The cells required exogenous OSKM TFs to maintain near-pluripotency, indicating that they were partially (or metastable) reprogrammed cells. Thus, we called them “iPS-like” cells. Through extensive genetic analysis, we confirmed that a line (designated “10-22” cells) was unambiguously derived from advanced PDAC cells because the cells harbored classical PDAC oncogenic mutations, such as mutations in the *KRAS* and *TP53* genes, a heterozygous deletion of *CDKN2A*, homozygous deletions of *SMAD4*, as well as chromosomal rearrangements of the original tumor epithelial cells. The 10-22 cells acquired pluripotency to some extent, yet preferentially generated PanIN2/3-like ductal lesions after three months in immunocompromised mice. The lesions progressed to the invasive stage by 6-9 months. Thus, cancer iPS-like cells replicated the natural course of human PDAC progression (Kim et al., 2013).

To identify secreted proteins from the early lesion, we isolated the PanIN lesions from mice transplanted with the 10-22 cells and cultured them as 3D organoids in serum-free media (Kim et al., 2013). Proteomic analysis of the filtered media derived from 10-22-derived PanINs successfully identified human proteins specific to the PanIN secreted proteome, which fell into RAS/P53/JUN/CTNB1 and TGFβ/integrin networks, as well as networks involving the transcription factor HNF4α, which had not been reported in PDAC. We confirmed that HNF4α expression was associated with various PanIN2, PanIN3, and well-differentiated PDAC, but not with poorly differentiated PDAC. Thus, the 10-22 iPS-like cells led to discovering a new gene network specific to the early-to -intermediate stages of PDAC (Kim et al., 2013).

We further validated a subset of these secreted proteins in three independent plasma cohorts derived from healthy subjects and patients with various stages of PDAC and benign diseases (Kim et al., 2017). THBS2 plasma levels identified early resectable stage I PDAC patients and all stages of PDAC patients from controls. Therefore, we have validated the application of our PDAC-iPS-like system as a tool for early diagnostic biomarker discovery (Kim et al., 2017).

Altogether, our proof-of-principle data demonstrated that the cancer reprogramming approach could provide a human cell model for unprecedented experimental access to the early stages of PDAC and serve as a new tool for discovering novel secreted proteins that can be used as candidate biomarkers.

Although PDAC is notably a challenge to reprogram, more recently, another study also showed reprogramming of PDAC. Khoshchehreh et al. attempted to reprogram multiple human PDAC patient-derived xenografts by introducing episomal vector-mediated OSK, Lin28A, LMYC, and *TP53* knockdown, and obtained a reprogrammed PDAC line (Khoshchehreh et al., 2019). Although this line appeared not to be fully reprogrammed, it lost invasiveness and sphere formation capacity *in vitro* and tumorigenicity *in vivo*. Thus, this study demonstrated that epigenetic reprogramming could decrease PDAC cells' aggressiveness and result in subsequent loss of *in vivo* tumorigenicity. However, the caveat is that this study did not assess the genetic status of reprogrammed PDAC or their parental PDAC cells. As PDAC consists of highly heterogeneous populations, it remains unclear if the observed loss of tumorigenicity was not due to the reprogramming of non-malignant cells from the original sample without PDAC-associated genetic lesions.

## 4 Molecular features of reprogramming of normal somatic cells

Efforts to understand the molecular mechanisms underlying OSKM reprogramming have been made in recent years, primarily using murine embryonic fibroblasts (MEFs), and have been reviewed in detail elsewhere (Buganim et al., 2013, Aydin and Mazzoni, 2019, Apostolou and Stadtfeld, 2018). Here, I highlight some of the key aspects of normal cell reprogramming that can inform cancer biology that will be subsequently discussed.

### 4.1 The trajectory of reprogramming

Successful reprogramming requires both erasure of the somatic cell identity and activation of the pluripotency gene program. The first phase of reprogramming involves rapid proliferation (Mikkelsen et al., 2008), suppression of somatic cell (fibroblast) markers (Soufi et al., 2012, Chronis et al., 168 (2017), Li et al., 2017), rewiring of the metabolome (Zhang et al., 2012, Folmes et al., 2011), induction of apoptosis (Soufi et al., 2012, Kim et al., 2018), and mesenchymal-to-epithelial transition (MET) (Cacchiarelli et al., 2015, Li et al., 2010, Samavarchi-Tehrani et al., 2010, Schiebinger et al., 2019). These initial reprogramming events occur in a stochastic manner (Hanna et al., 2009, Buganim et al., 2012) and produce intermediate reprogrammed cells (Buganim et al., 2012). This is immediately followed by a “hierarchical” phase where a small fraction of the intermediate cells are fully reprogrammed into iPSCs by reactivating an endogenous pluripotency gene network (Buganim et al., 2012, Polo et al., 2012, Knaupp et al., 2017). This process is considered an inefficient and rate-limiting process due to barriers attributed to refractory genome states (Soufi et al., 2012, Buganim et al., 2012).

### 4.2 Role of reprogramming TFs in cellular reprogramming

Despite the low efficiency of reprogramming and attempts to replace OSKM factors with alternatives, enforced OSKM expression provides the most robust reprogramming efficiency thus far. What is the role of these reprogramming factors in cellular reprogramming?

It is clear that OSKM induction triggers rapid genome-wide changes in chromatin before gene activation, and such chromatin remodeling is dynamic throughout the entire process (Li et al., 2017, Knaupp et al., 2017, Koche et al., 2011, Hussein et al., 2014). For example, in the early reprogramming of MEFs, rapid genome-wide changes predominantly occurred in the euchromatic histone modification H3K4me2 at many loci, which included large subsets of pluripotency-related or developmentally-regulated gene promoters and enhancers (Koche et al., 2011). Another study also showed dynamic changes in chromatin accessibility of isolated MEF sub-populations of reprogramming intermediates during reprogramming (early accessible sites that gain accessibility shortly after OSKM induction and remain open throughout the iPS state, transient accessible sites that are early accessible sites reverting to inaccessible by the iPSC state, late accessible sites that gain accessibility in iPSC state, inaccessible site that loss accessibility during reprogramming). Notably, half of the early accessible sites that transited from inaccessible sites and around 90% of the transiently accessible sites in MEF reprogramming overlapped with sites bound by OCT4/SOX2, while OCT4/SOX2 drove late accessible sites to a lesser extent (Knaupp et al., 2017).

Does ectopic OSKM expression change chromatin structure directly? Various studies showed the causative effect of ectopic OCT4 expression on the reorganization of chromatin structure. One study showed that ectopic OCT4 competed with relatively unstable nucleosomes and subsequently established nucleosome-depleted regions in the regulatory regions of target genes in hypomethylated clones derived from the colon cancer line HCT 116 (You et al., 2011). Another study showed that exogenous OCT4 bound to the nucleosome-depleted areas of NYOD1 enhancers flanked by nucleosomes marked by the poised or permissive enhancer mark H3K4me1. This OCT4 binding allowed promoters to convert from repressive H3K27me3 marks to the bivalent mark H3K27me3 and H3K4me3, representing a poised state of developmental genes in ES cells (Taberlay et al., 2011). KLF4 binding was shown to require the de novo establishment of enhancer and promoter contacts within specific enhancer hubs (Di Giammartino et al., 2019).

How does ectopic OSKM expression initiate a new gene program? OCT4, SOX2, and KLF4 are widely known to function as pioneer factors (i.e., they target distal enhancers of silent genes within closed chromatin to endow competence for gene reactivation), especially in human fibroblasts (Soufi et al., 2012, Knaupp et al., 2017, Narayan et al., 2017). In contrast, MYC is known to preferentially bind to active promoters and cooperatively enhance occupancy of OSK in both human and mouse (Soufi et al., 2012, Chronis et al., 168 (2017), Knaupp et al., 2017). Interestingly, in MEFs, unlike in human cells, collaborative OSK was required to open a subset of pluripotent enhancers (Chronis et al., 168 (2017)). Mechanistically, OCT4, SOX2, and KLF4 are shown to bind to one face of the DNA helix with a partial motif, thus allowing a nucleosome to bind to the other side of DNA (Soufi et al., 2015). Moreover, two studies showed that OCT4 was capable of binding to methylated regions in murine ES cells (Yin et al., 2017) and during reprogramming of MEFs (Knaupp et al., 2017), although this conflicted with a previous report (Chen et al., 2016). Thus, this pioneering function can explain in part how OSKM can initiate new cell lineage programs, such as pluripotency (Iwafuchi-Doi and Zaret, 2014).

Additionally, OSKM expression has been shown to erase the original cell identity directly or indirectly (Chronis et al., 168 (2017), Cacchiarelli et al., 2015, Takahashi et al., 2014, Buganim et al., 2012, Polo et al., 2012, Knaupp et al., 2017). For example, in purified intermediate cells during MEF reprogramming, OCT4/SOX2 redistributed somatic TFs from somatic enhancers to transiently accessible regions that they engaged (Knaupp et al., 2017). In another study of bulk MEFs during reprogramming, OSK induced the redistribution of somatic TFs away from MEF enhancers to sites engaged by OSK elsewhere in the genome, which led to global destabilization of MEF enhancers (Chronis et al., 168 (2017)). On the other hand, OSKM suppressed essential somatic genes indirectly by recruiting either the SIN3 co-repressor complex (Li et al., 2017) or Hdac1 to MEF enhancers engaged by OSKM (Chronis et al., 168 (2017)).

In summary, OSKM induction is accompanied by highly dynamic chromatin remodeling to initiate cellular reprogramming of fibroblasts to iPSCs. These chromatin dynamics translate into distinct phenotypes at the very early and late stages, as well as in reprogramming intermediates that may follow diverse paths through transient states.

## 5 Parallels between pluripotency and cancer

### 5.1 Cellular reprogramming vs. cancer development

The sustained expression of the reprogramming TFs in normal cells - either individually or in combination – is known to contribute to tumorigenesis. For example, the inducible expression of OCT4 in mice initiated dysplasia by preventing the differentiation of multiple lineages (Hochedlinger et al., 2005). KLF4 is overexpressed in human PDAC, and ectopic KLF4 expression was sufficient to induce cells to undergo acinar ductal metaplasia (ADM) in the presence of mutant *Kras* in mice (Wei et al., 2016). SOX2 was the most upregulated TF (Boumahdi et al., 2014) in cancer stem cells from skin squamous cell carcinoma (SCC) and was shown to control self-renewal (Sastre-Perona et al., 2019).

Partially reprogrammed cells have also been shown to display cancerous phenotypes (Knappe et al., 2016, Nishi et al., 2014, Shibata et al., 2018, Ohnishi et al., 2014). For instance, while fully reprogramed mice (*Rosa26*-rtTA; TetO-OSKM) displayed teratomas (Abad et al., 2013), partially reprogramed mice developed poorly differentiated tumors through altered epigenetic regulation (Ohnishi et al., 2014). However, these distinct phenotypes can also be attributed to the different transgenic systems used to induce TetO-OSKM in two studies (germline-transmitted transgenic mice vs. chimeric mice with transgenes in the collagen locus). Ohnishi et al. showed that prolonged (>7 days) activation of OSKM by doxycycline resulted in the development of poorly differentiated invasive tumors in multiple mouse organs after doxycycline withdrawal (Ohnishi et al., 2014). The dox-withdrawn tumors showed loss of somatic cell identity and shared gene signatures of ES-Core and MYC modules (Kim et al., 2010) with murine ES cells (Ohnishi et al., 2014). PRC represses a large cohort of developmental regulators in ES cells to maintain pluripotency (Boyer et al., 2006, Lee et al., 2006). Intriguingly, the ES PRC module (Kim et al., 2010) was differentially expressed in the tumors than in the ES cells. Specifically, a number of ES-PRC-targeted genes were not repressed in the dox-withdrawn tumor (Ohnishi et al., 2014). This suggests that failed PRC activation in partially reprogrammed mice may be associated with the activation of a developmental gene program, which may be compatible with tumor development.

Shibata et al. showed that transient OSKM expression induced reversible downregulation of acinar cell-related genes by partly inhibiting H3K27Ac deposition at the enhancers of several acinar-related genes and caused a loss of acinar cell identity in the pancreas (Shibata et al., 2018). Moreover, OSKM induction, along with *Pdx1*-Cre activation of *Kras* and *p53* mutations, resulted in widespread PDAC development (Shibata et al., 2018), suggesting that OSKM-mediated destabilization of somatic cell identity catalyzes irreversible cancer development.

Altogether, these results suggest that OSKM-mediated reprogramming, which triggers a rewiring of the starting cells' epigenetic status, shares molecular routes with cancer development. Many master TFs involved in cellular reprogramming and trans-differentiation have been shown to have a pioneering function (Soufi et al., 2012, Soufi et al., 2015, Fernandez Garcia et al., 2019) and can thus initiate regulatory events at particular sites in chromatin. Such pioneer TFs are aberrantly dysregulated in various cancers (Iwafuchi-Doi and Zaret, 2014, Roe et al., 170 (2017)). Thus, it is not surprising that the cellular reprogramming process mimics cancer development, at least in part. The similarity between cellular reprogramming and cancer development may raise some concerns against harnessing cellular reprogramming to study cancer.

### 5.2 iPSC vs. cancer

Undoubtedly, ES cells and cancer cells share some properties, including self-renewal and indefinite proliferation (Kim et al., 2010). However, unlike cancer cells, ES cells maintain well-coordinated intrinsic mechanisms to control their proliferation (Hendrix et al., 2007, Gonzales et al., 2015), differentiate into the three germ layers, and form benign teratomas. In contrast, cancer cells do not have any of these properties.

Does reprogramming into pluripotency allow cancer cells to acquire such abilities? Let's compare the two endpoint products of each process: iPSCs vs. cancer cells. Doi et al. suggested parallel mechanisms of epigenetic reprogramming between iPSCs and cancer by investigating differentially methylated regions (DMR) in reprogrammed cells vs. their parental fibroblast cells (R-DMR) and in cancer cells vs. their matched normal cell types (C-DMR) (Doi et al., 2009). Hypomethylated R-DMRs were associated with hypermethylated C-DMRs, and, conversely, hypermethylated R-DMRs were associated with hypomethylated C-DMRs (Doi et al., 2009). This suggests that aberrantly methylated regions in cancer could be reset during reprogramming. Indeed, reprogramming studies with GBM-GNS (Stricker et al., 2013) and sarcoma (Zhang et al., 2013) showed global DNA demethylation during reprogramming.

Notably, all three factors (OCT4, SOX2, and KLF4) were required to reprogram cancer cells and reduce their tumorigenicity in many studies (Zhang et al., 2013, Kim and Zaret, 2015, Kim et al., 2013, Khoshchehreh et al., 2019, Miyoshi et al., 2010). Overexpression of single reprogramming TFs in cancer failed to reprogram cancer cells and made them more aggressive, even though the original cancer cells expressed some of the TFs endogenously (Kim and Zaret, 2015, Kumar et al., 2012). This indicates that the cooperative effects of OSKM are crucial to reprogram cancers.

In summary, although initial cellular reprogramming is reminiscent of cancer development, pluripotent stem cells have an epigenetic landscape that is distinct and reciprocal to that of cancer cells. Moreover, several cancer iPSC studies have provided evidence of the rewiring of part of the cancer epigenome as a result of reprogramming. This raises exciting questions: Can cellular reprogramming offer a powerful tool to deconstruct and reconstruct aberrant cancer epigenomes? Does it provide a new way to tackle cancer as well as serve as a cancer progression model?

## 6 Perspective: What can we learn from cancer reprogramming?

### 6.1 Cancer reprogramming to disrupt the cancer epigenome

We now know that OSKM can cooperatively repress somatic enhancers, which at least in part, erases the original cellular identity at the beginning of fibroblast reprogramming (see section 3). The OSKM factors can also reorganize chromatin structure before the activation of pluripotency. It remains to be determined if these mechanisms occur during the reprogramming of cancer cells. A growing number of studies show the potential of cellular reprogramming to model cancers. Yet, it remains unclear how the OSKM TFs engage the cancer genome and begin erasing the cancer epigenome to guide the reversion of cellular identity back to a pluripotent state. Common epigenetic factors can differentially elicit tumor formation or reprogramming to pluripotency. Why is it necessary to study early reprogramming events in cancer?

First, reprogramming TFs can change the chromatin structure of the original cells with pioneering functions as well as by recruiting other chromatin co-factors. OCT4/SOX2 can redistribute somatic TFs to the transiently accessible regions by OCT4/SOX2 (Knaupp et al., 2017) or directly toward new sites that include pluripotency enhancers (Chronis et al., 168 (2017)). This is a potential mechanism by which TFs can silence the somatic transcriptional network. Aberrant transcriptional networks governed by master TFs are seen in various cancers (Iwafuchi-Doi and Zaret, 2014). For instance, pioneer TF FOXA1 is reported to reprogram murine metastatic PDAC enhancers (Roe et al., 170 (2017)). De novo PITX1 expression in tumor-propagating cells controlled self-renewal and proliferation through co-binding with SOX2 and TRP63 and repressing KLF4, which maintains differentiation, in murine SCC (Sastre-Perona et al., 2019). Thus, an interesting question is whether OSKM induction can redistribute such aberrantly activated cancer-specific master TFs and disrupt the cancer epigenome. Such findings may point to new ways to tackle cancers.

**Fig. 1:**
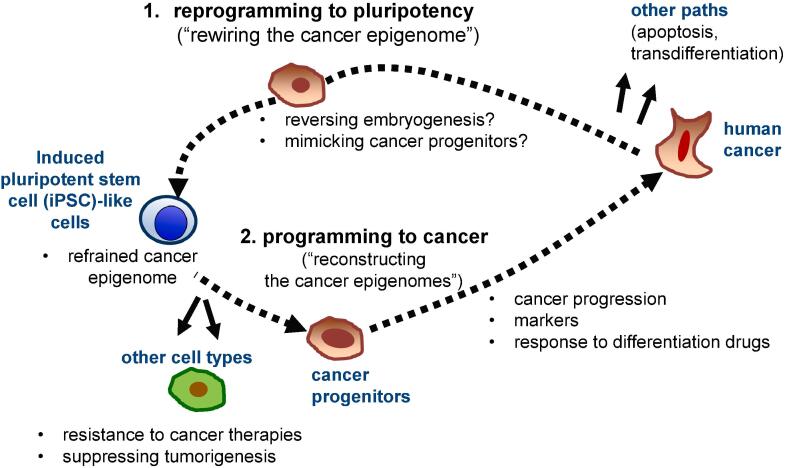
**Lessons from cancer reprogramming**. A prospective scenario for (1) rewiring and (2) reconstructing the epigenome during reprogramming into iPSCs and differentiation into cancer lineages, respectively. In this way, cancer reprogramming can serve as a useful tool to unveil epigenetic alterations determined by genetic mutations.

Second, malignancy can be manifested in cells in which oncogenic pathways can be active. Not all cell types harboring the same mutations can develop cancer. Reprogramming to pluripotency or lineage-transdifferentiation by master TFs provides a way to change cellular identity. Reprogramming has been shown to disrupt oncogenic addiction and result in resistance to therapy. Likewise, transdifferentiation also results in decreased malignancy in a subset of tumors. For example, transdifferentiation of B lymphomas into macrophage-like cells impaired tumorigenicity in xenograft hosts (Rapino et al., 2013, McClellan et al., 2015). Reintroducing acinar TF PTF1A into established PanINs reverted them to quiescent acinar cells in the murine PDAC model (Krah et al., 4 (2015).), and overexpression of PTF1A inhibited PDAC growths in human PDAC cell lines (Krah et al., 2019). Therefore, elucidating the mechanism by which reprogramming or transdifferentiating master TFs begin rewiring the cancer epigenome may give insights into the molecular basis of modulating the cancer phenotypes (Fig. 1).

### 6.2 Cancer reprogramming to reconstruct the cancer epigenome

The evolution of cancer has not been well characterized in many solid tumors, in part due to the lack of appropriate models. Current models for the study of solid human tumors recapitulate advanced stages of tumors and do not reflect the events that occur early in the disease's progression. Reversible epigenetic alterations can play a crucial role in the progression of cancers. Such epigenetic changes that accumulate in cancer cells can be reset to some extent during reprogramming to pluripotency by NT in murine tumors (Blelloch et al., 2004, Hochedlinger et al., 2004, Li et al., 2003) or by iPSC technologies in human cancers (Kim and Zaret, 2015, Papapetrou, 2016).

In embryogenesis, each cell of the blastocyst's inner cell mass must remain in the ground state of pluripotency until implantation (Nichols and Smith, 2012). Oncogenic pathways are spatially and temporally well-regulated during embryogenesis (Kim and Zaret, 2015). ES cells, similarly to the pre-implantation stage blastocyst, can restrict uncontrolled cell growth in the pluripotent state and keep the balance between self-renewal and differentiation (Hendrix et al., 2007, Gonzales et al., 2015). Early studies showed that cancer stem cells resemble undifferentiated cells of the early post-implantation stage rather than pre-implantation stage cells in their structure (Damjanov et al., 1971) and alkaline phosphatase content (Damjanov et al., 1971). Taken together, these imply that the pluripotent environment can be dominant over the cancer phenotype.

However, upon release from pluripotency, the rewired epigenetic landscapes can be re-established in the presence of the genetic mutations and cellular specifications that originally contributed to the cancer transformation. While NT-reprogrammed cancer cells differentiated into multiple early developmental cell types of the embryo, pediatric tumor-derived embryos died partly through organogenesis (Blelloch et al., 2004, Li et al., 2003). In contrast, RAS-inducible mouse melanoma cells reprogrammed to pluripotency by NT contributed to animal tissues in chimeric mice and ultimately gave rise to melanomas and rhabdomyosarcomas, which share oncogenic pathways with melanoma (Hochedlinger et al., 2004). This indicates that oncogenic pathways can be reactivated by irreversible genetic alterations during organogenesis (McKinnell et al., 1969, Blelloch et al., 2004, Hochedlinger et al., 2004, Li et al., 2003). Thus, reprogramming of cancer cells to pluripotency and subsequent programming back to their original cellular state can allow us to study the dynamic events in the course of disease progression (Chao et al., 2017, Kotini et al., 2017, Kotini et al., 2015, Kim et al., 2013, Kim and Zaret, 2015, Papapetrou, 2016, Stricker et al., 2013, Zhang et al., 2013, Lee et al., 2015, Bernhardt et al., 2017). Therefore, cellular reprogramming of cancer cells could offer a useful tool to unveil cancer evolution driven by epigenetic alterations or aberrant transcriptional gene networks in the presence of mutations (Fig. 1).

## 7 The challenges to overcome

To answer these provocative questions, we need to overcome a few challenges in the next few years. The biggest obstacles in cancer reprogramming mainly arise from the fact that tumors are highly heterogeneous, yet only a subset of cells are reprogrammed. Genetic mutations could be barriers impeding the reprogramming process. Most of the reprogramming mechanistic studies have been done in normal fibroblasts or blood cells, and the cumulative information from such cell types may not apply to cancer cells.

### 7.1 Normal cell contamination

Solid tumors consist of cancer cells mixed with non-neoplastic cells. When it comes to human PDAC, this is a more severe problem since PDAC epithelial cells are tightly surrounded by stromal cells. We found that 90% of tumor epithelial cells did not harbor *KRAS* mutant alleles (unpublished data). Considering that the efficiency of reprogramming is extremely low and the reprogramming process is a stochastic event, it is not feasible to select cancer iPSC clones harboring the given genetic alteration. We were only able to identify one iPS-like cell line harboring PDAC classical mutations after multiple attempts. In the case of blood cancers, this may be less of a challenge as blood cells can easily be separated from stroma, and indeed the most significant advances in the cancer iPSC field have been made in blood cancer. Nonetheless, blood cancer samples still contain non-neoplastic cells, thus it is always crucial to confirm the origin of iPSCs.

Before introducing reprogramming TFs, the heterogeneous tumors should be purified using known surface markers to enrich cancer cells. Second, unlike normal fibroblast reprogramming, an additional labor-intensive step of picking as many colonies as possible is required. Finally, the most critical step is to ascertain the origin of iPSCs through extensive genetic studies of the starting normal and cancer genome along with the normal and cancer iPSCs. For example, for an initial quick screen of cancer clones, our study quantified mutant *KRAS* alleles by pyrosequencing and then confirmed the origin of iPSCs by comparative genome hybridization (CGH) (Kim et al., 2013). Today, due to reduced cost and improved resolution, whole-exome sequencing has replaced CGH. Droplet Digital PCR (ddPCR) (Hindson et al., 2011) also allows to detect and quantify somatic mutation frequency. Both pyrosequencing and ddPCR detection have limits as low as 3%-5%. Thus, if there is a set of specific mutations known to recur in a given cancer, pyrosequencing or ddPCR can be used to quickly screen colonies as well as to quantify the fraction of cancer cells in a primary tumor at low cost. Once a few colonies are verified, they, along with the parental cells, should be examined with deep sequencing to confirm the clones' origin and detect any other additional de novo mutations.

### 7.2 Intratumoral and Intertumoral heterogeneity

Intratumoral heterogeneity can give rise to subclones, which may differ in reprogramming efficiency. A stochastic model of reprogramming posits that cells have equal potential to give rise to iPSCs with variable latency at the single-cell level (Hanna et al., 2009). However, a recent study using mathematical modeling and a DNA barcoding strategy in MEFs showed that a subset of cells could be preferentially reprogrammed at the clonal level (privilege model) (Shakiba et al., 2019). The privilege model may be more relevant to cancer because cancer cells may exhibit aberrant genetic, epigenetic, and metabolic pathways. Even if all cancer cells in a given tumor have the same genetic alterations, not all cancer cells have the same epigenetic and metabolic changes. Thus likely not all cells in a tumor have the same potential to be reprogrammed. Exploiting subclone-specific, promoter-driven markers or barcodes, in combination with extensive genetic screening and histological analysis, could address this issue.

Since there is significant variability among patients, generating iPSCs from multiple patients is needed. It is also essential to validate the significant results obtained from cancer iPSCs in more patient samples by other methods. Our study validated a subset of biomarkers generated from a single-iPS-like cell line in multiple patient cohorts (Kim et al., 2017). Intertumoral heterogeneity also complicates bioinformatic analyses. We found that it was not feasible to use PDACs from different patients as biological replicates. Hence, a better approach would be to analyze each patient sample separately and validate the findings in other tumors (modeling and validation cohorts). Single-cell analysis might be another option with pseudo-bulk analysis regarding each cell as one replicate.

### 7.3 Low reprogramming efficiency: epigenetic memory and genetic mutation/aneuploidy

Both NT and iPSC studies show the low reprogramming efficiency of cancer cells. In contrast to most myeloid leukemia iPSC studies that documented complete removal of somatic identity in iPSCs, solid cancer iPSC studies show persisting epigenetic changes that prevent cells from being fully reprogrammed pluripotency (Kim et al., 2013, Khoshchehreh et al., 2019, Zhang et al., 2013, Bernhardt et al., 2017). Paradoxically, this low reprogramming efficiency provided an opportunity to develop a human model of cancer progression (Kim et al., 2013).

Cells with certain mutations are resistant to reprogramming (Lee et al., 2017, Munoz-Lopez et al., 2016, Kim and Zaret, 2015), and reactivation of the DNA repair machinery by reprogramming was shown to rescue chromosomal abnormalities in iPSCs in a cell-autonomous manner (Kotini et al., 2015, Bershteyn et al., 2014). Also, PDAC pretreated with radiotherapy could not generate any iPS-like clones in our studies, suggesting that radiation-induced senescence may have impaired reprogramming (Kim and Zaret, 2015). Furthermore, aneuploidy can cause several cellular stress responses, including the activation of p53 through stress kinase p38 (Thompson and Compton, 2010). Tetraploid murine melanoma cell NT-ES cells failed to produce teratomas and chimeras (Hochedlinger et al., 2004). Likewise, human aneuploid cancer cells could not activate muscle genes when fused with muscle cells unless cells were pretreated with an inhibitor of DNA methylation (Chiu and Blau, 1985). These data indicate that aneuploidy or certain genetic alterations could impede TF-mediated cancer reprogramming. As a result, reprogramming per se may be selecting non-malignant cells.

## 8 Closing remarks

Despite the caveats and challenges to overcome, a handful of proof-of-principle cancer reprogramming studies have provided new insights into disease progression, cancer epigenetics, and cancer therapy. By better understanding cellular reprogramming of cancer cells, we may offer new opportunities to model and understand neoplastic diseases.

## Declaration of Competing Interest

The authors declare that they have no known competing financial interests or personal relationships that could have appeared to influence the work reported in this paper.
